# Separating Gold, Silver and Copper Alloy by Electrolysis

**Published:** 1881-05

**Authors:** 


### SEPARATING GOLD, SILVER, AND COPPER ALLOY BY ELECTROLYSIS.

Among the later inventions well worthy of the attention of metallurgists, is the electrolytical process of refining, patented by E. Andre, both in this country and in Europe. This process has not been practically applied in this country, but is said to be in operation at the copper works of Messrs. Mason & Elkington, Pembrey, South Wales; at Birmingham and Manchester, England; the government works at Mahsfeld, Ocker, and Duisburg, Germany, and the refining works at Hamburg and Frankfort-on-the-Main. The results obtained at these works are said to be of the most satisfactory nature, and with the improvements of the dynamo-electro machine, which are continually making, even better results are expected in the future. This process is said to completely extract, more economically than by other means, the precious metals from their base alloys, and separate the latter in a chemically pure metallic form. Mr. Andre employs the current of the dynamo-electric machine on the material to be separated as anodes suspended in diaphragms in a diluted acid or alkaline bath, according to circumstances. When the disintegration of the alloys occurs, the precious metals are retained in the diaphragms, and the baser metals are deposited in a pure metallic state upon the cathodes placed opposite the anodes. The bath is changed at proper times, to free it from accumulated impurities, such as zinc, iron, antimony, etc.—*Engineering and Mining Journal.*

